# Analysis of farmland fragmentation in China Modernization Demonstration Zone since “Reform and Openness”: a case study of South Jiangsu Province

**DOI:** 10.1038/srep11797

**Published:** 2015-07-02

**Authors:** Liang Cheng, Nan Xia, Penghui Jiang, Lishan Zhong, Yuzhe Pian, Yuewei Duan, Qiuhao Huang, Manchun Li

**Affiliations:** 1 Jiangsu Provincial Key Laboratory of Geographic Information Science and Technology, Nanjing University, Nanjing, 210093, China; 2 Collaborative Innovation Center for the South Sea Studies, Nanjing University, Nanjing 210093, China; 3 Department of Geographic Information Science, Nanjing University, Nanjing 210093, China; 4 Collaborative Innovation Center of Novel Software Technology and Industrialization, Nanjing University, Nanjing, China

## Abstract

Farmland is a fundamental resource for human survival and development. However, farmland fragmentation has become a serious problem, causing ecological damage and low crop production efficiency in many parts of the world. Based on remote sensing and socioeconomic data, we used landscape pattern indices, Morphological Spatial Pattern Analysis (MSPA), and Markov chain models to analyze the temporal and spatial pattern changes in farmland in South Jiangsu Province (the first “Modernization Demonstration Zone” in China) during 1985–2010. Our results demonstrated that the total farmland area decreased by ca. 24% and the farmland pattern became fragmented during 1985–2008: core farmland decreased and islet farmland increased. Additionally, the farmland patch density (*PD*) increased and three other landscape indices (*NLSI*, *MESH*, and *COHESION*) showed significant decreases. Although the fragmentation rate slowed after 2008, the convergence rate to a stationary farmland distribution became faster, and transitions tended to be less deterministic after 2000. Economic and population growth and policy changes positively contributed to this phenomenon. Therefore, the primary task of farmland protection should be to preserve contiguous farmlands and reduce scattered patches in order to promote farmland connectivity.

Farmland is the basis of food and economic security, the foundation of agricultural civilization^1^, and a vital habitat for several ecosystems^2^. Thousands of years of agricultural activities have made farmland one of the largest terrestrial ecosystems, meeting the needs of a constantly growing population^3^. However, since the 1960s, as the population has increased and agricultural technology has improved^4^,^5^, there has been a global drop in the total farmland area and the per capita farmland availability^6^. This loss of farmland is predominantly due to anthropogenic activities, such as occupation (the replacement of farmland) by road developments and urban sprawl, and these factors may also cause physical fragmentation of farmland landscape^7^,^8^.

In landscape ecology, physical fragmentation is defined as the breaking up of a habitat, ecosystem or land-use type into smaller independent patches^9^,^10^. Farmland fragmentation can influence biodiversity and ecosystem functions^11^, and can cause aesthetic and hydrological damage^12^. It also hinders centralized management and large-scale farmland operations^13^. The elimination of fragmentation, by means such as farmland consolidation, could potentially increase the effective farmland area, increase food production, and reduce the labor cost of food production^14^. Farmland connectivity is essential for the economic viability of rural areas, urban growth^15^, maintaining biodiversity and habitat heterogeneity^16^,^17^.

A number of studies have used landscape pattern indices^8^,^18^,^19^ or spatial autocorrelation indices in the GIS (Geographic Information System)^20^ to explore farmland landscape structures and the driving forces of fragmentation. These traditional index methods focus on numerical analysis of fragmentation^21^, so a new graphical Morphological Spatial Pattern Analysis (MSPA) method was introduced^22^. MSPA can objectively identify and map landscape structures, such as corridors (connectors) which are essential in landscape ecology^23^. In recent years, MSPA has been successfully applied to analyzing morphological changes in different landscapes, including forest^23^,^24^, green infrastructure^25^, and wetland^26^. However, applications of MSPA to China’s farmlands are rare and few studies exist on landscape pattern changes^27^. China, the largest developing country, has undergone tremendous changes in many fields since the “Reform and Openness” beginning in 1978. The most conspicuous change is urbanization, which has directly and deeply influenced China’s vast rural areas^28^,^29^. However, excessively rapid urbanization and economic development cause significant rural population loss, ecological damage^30^,^31^, and permanent farmland loss^32^, which directly cause farmland fragmentation. Although the government has implemented strict farmland protection systems to preserve high-quality and contiguous farmlands^33^, fragmentation has remained high in China since the 1990s^34^. Consequently, research on farmland pattern changes is urgently needed.

In China, farmland fragmentation is apparent in the southeast coastal region^35^, particularly in South Jiangsu Province. Although South Jiangsu Province has favorable farming conditions, common farmland problems are still relevant, alongside urbanization. We combined remote sensing (RS), landscape pattern indices, MSPA, and a Markov chain model for this study. The primary aims of this study are to (1) present the spatial and temporal changes of farmland landscape fragmentation in South Jiangsu Province at multiple scales, (2) identify the rate of change and the transition trends for farmland, and (3) analyze the driving forces behind farmland fragmentation and inform farmland protection policies.

## Results and Discussions

### Farmland changes

Our classification results (Table 1) demonstrate that the total farmland area in South Jiangsu Province decreased from 17915 km^2^ to 13638.64 km^2^ during 1985–2010. The net reduction is about 4300 km^2^ and the total proportion of farmland decreased from 64.56% to 49.10%. The average annual drop in farmland area increased from 95.56 km^2^ (1985–1995) to 389.96 km^2^ (2005–2008). Similarly, the average annual decrement rate increased from 0.53% (1985–1995) to 2.57% (2005–2008) before 2008. However, the average annual drop and decrement rate slowed during 2008–2010, falling to 193.09 km^2^ and 1.38%, respectively.

### Farmland fragmentation at the landscape level

**Figure 1:**
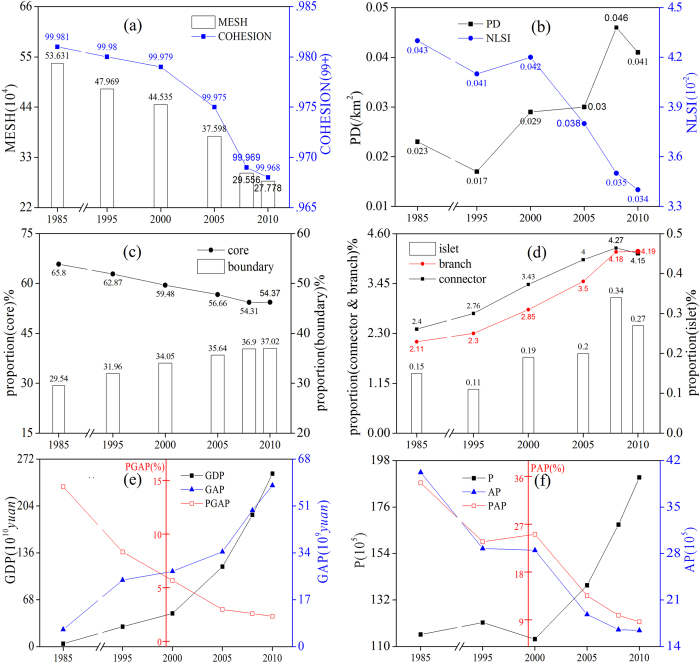
Landscape index changes (**a**,**b**; top) for farmland, including the patch density (*PD*), the normalized landscape shape index (*NLSI*), *COHESION*, and the effective *MESH* size (*MESH*). Changes in the proportion of different MSPA types for the entire farmland area (**c**,**d**; middle). Changes in the number of practitioners (*P*), agricultural practitioners (*AP*), the relative proportion of *AP* (*PAP*), the regional Gross Domestic Production (*GDP*), the regional Gross Agricultural Production (*GAP*) and the proportion of *GAP* (*PGAP*) (**e**,**f**; bottom) in South Jiangsu Province during 1985–2010. The figure was generated by L.Z. and Y.P. using Origin 8.5 (http://www.originlab.com/).

The *PD* of farmland landscape (Fig. 1a,b) in study area generally increased during 1995–2008, and decreased slightly during 1985–1995 and 2008–2010. The *NLSI*, *COHESION* and *MESH* indices, decreased synchronously during 1985–2010. The reduction rate for these three indices increased after 2000, but eased off after 2008. The change in the *PD*, *COHESION* and *MESH* indices reflected that farmland fragmentation became more significant. The *NLSI* was very low (<0.1) during 1985–2010, indicating that the type of farmland shape was relatively rare. In summary, these four indices suggested increased fragmentation of the farmland landscape in South Jiangsu during 1985–2010.

### Farmland fragmentation at the pixel level

The changes in the relative proportions of the five MSPA types (made up of seven classes, see Methods and Table 2 for details) in this area show the progress of farmland fragmentation (Fig. 1c,d). During 1985–2010, the proportion of core farmland (representing the main stable and contiguous farmland) decreased from 65.8% to 54.37%. The boundary (perforation and edge classes) farmland increased from 29.54% to 37.02%, which implies that the core farmland acquired a more irregular shape. Overall, the relative proportion of islet farmland (interspersed farmland unsuitable for mass production) almost doubled, which directly reflected the increased fragmentation. Although the proportion of connectors (loop and bridge classes) increased from 2.4% to 4.2%, demonstrating the improved connectivity between core farmlands, the simultaneous increment (from 2.11% to 4.19%) of branch farmland (randomly attached to boundary farmland) indicated that the fragmentation was still increasing.

**Figure 2:**
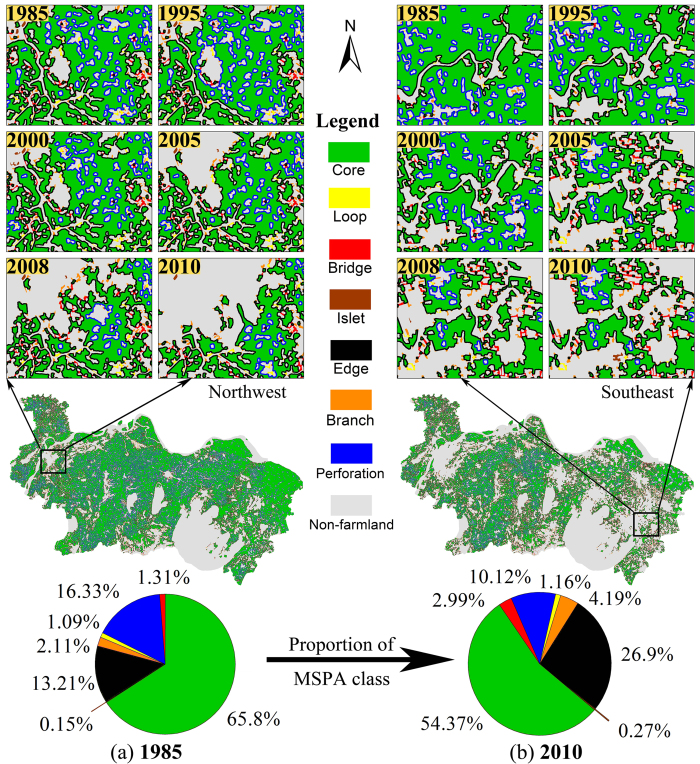
Map of MSPA classes and the relative proportion of each MSPA class for (**a**) 1985 and (**b**) 2010. The figures shows maps of MSPA class changes during 1985–2010 in two of the most fragmented small regions (located in northwest and southeast of South Jiangsu Province). The map data was downloaded from http://www.geodata.cn (Administrative Boundaries of South Jiangsu Province). The figure was generated by N.X. and P.J. using ArcMap 10.0 (http://www.esrichina.com.cn/) and Adobe Photoshop CS5 (http://www.adobe.com/cn/products/photoshop.html).

In addition to the results presented above, MSPA allows for direct observation of farmland fragmentation, as shown in Fig. 2, which clearly shows that farmland area has sharply deceased, and become irregular and fragmented. The majority of fragmented farmland in the study area (Fig. 2) were in the southeast, close to Shanghai (the economic and financial center of China), and in the northwest near Nanjing (the capital of Jiangsu Province). These two cities affect the surrounding regions significantly and directly influence the farmland configuration in South Jiangsu Province.

### Markov chain models for MSPA classes

**Figure 3:**
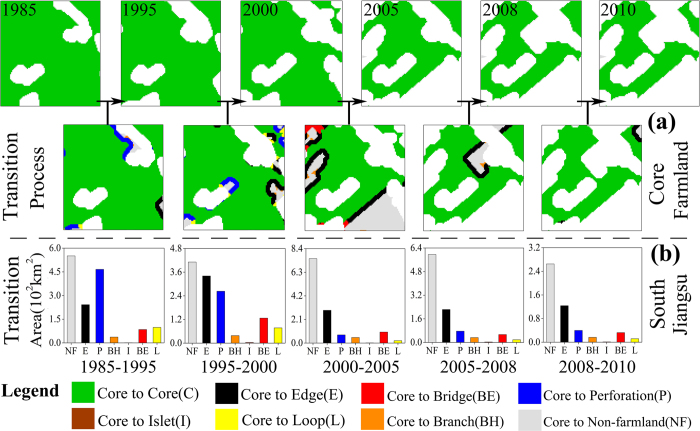
Farmland transitions: (**a**) An example of transitions in a small region of core farmland in the study area; (**b**) The transitions from core farmland to other farmland classes for the entire South Jiangsu Province during 1985–2010. The figure was generated by N.X. and P.J. using GUIDOS (Vogt P, 2014. GuidosToolbox (Graphical User Interface for the Description of image Objects and their Shapes): Digital image analysis software collection available at the following web site: http://forest.jrc.ec.europa.eu/download/software/guidos) and Adobe Photoshop CS5 (http://www.adobe.com/cn/products/photoshop.html).

By applying a Markov chain model to the MSPA results, we can see the conversions between different morphological classes before and after 2000 from the transition probability matrices (Supplementary Table S1, S2). We can also map the transition process of core farmland in a small region (Fig. 3a), and the transition areas where core farmland was converted to other classes across the entire South Jiangsu (Fig. 3b). During 1985–2010, transitions mainly occurred within the same class, as shown by bigger diagonal values (>0.5) in the transition probability matrix. Core farmland was the most stable part, with about 80% remaining unchanged, which shows the stability of large continuous farmlands. Transitions between different classes (but within the same type) mainly occurred from the perforation to edge class (boundary type), and from the loop to bridge class (connector type), as shown by the larger off-diagonal values: transition probabilities were 0.1816 and 0.1861, respectively, before 2000, and 0.2690 and 0.1791 after 2000. These two transitions clearly show the farmland fragmentation from the sides (Supplementary Figure S1). Almost 75% of transitions to non-farmland occurred from islet and branch farmland, but transitions from non-farmland to farmland were extremely rare. Apart from branch and islet farmland, almost every farmland class appeared more stable before 2000. These results combine to indicate a more fragmented and poorly connected scenario after 2000.

The convergence rate (*ρ*) and normalized entropy (H(**P**)) of the transition matrix during 1985–2000 was 1.1647 and 0.4093, respectively. For the next decade (2000–2010), *ρ* was 1.2256, which indicates a faster convergence rate to a stationary farmland distribution, and more frequent transitions between different classes. H(**P**) for this decade was 0.3848, which indicates less deterministic and more unpredictable Markov transitions compared with the previous period. H(**P**) for 1985–2010 was approximately 0.39, indicating a partially deterministic transition process. The larger convergence rate and smaller entropy after 2000 indicate that the farmland fragmentation rate increased and was not well controlled.

### Driving forces behind farmland loss and fragmentation

Although correlations between the chosen factors (see Methods), islet farmlands and the *PD* were not significant, significant correlations were seen between factors, and other classes and indices, indicating the appropriateness of our chosen factors (Table 3). The regional *GDP* grew from 43.3 billion Yuan (the monetary unit of China) to 2506.74 billion Yuan during 1985–2010, while the regional *GAP* grew from 6.24 billion Yuan to just 58.43 billion Yuan (Supplementary Table S3, Fig. 1e,f). The proportion of *GAP* decreased from 14.43% to 2.33%, accompanied by a significant loss of local agricultural practitioners caused by the regional South Jiangsu Model^36^(a kind of economic development model which relied mainly on a collective economy). The economic barycenter transferred to the secondary and tertiary industries, causing significant loss and fragmentation of farmland.

Our results also showed different farmland loss and fragmentation rates during 1985–2010 (Figs 1,2). The rate was slow during 1985–1995, sharply increased after 1995, but slowed down after 2008. Combined with related policies, we found that China had been restoring the national economy from the beginning of the “Reform and Openness” program up to the late 1980s. Since 1992, China has established a socialist market economic system, and has begun to develop the economy energetically. South Jiangsu Province has attracted investment and developed township enterprise as a result of grants from the “Reform and Openness”; however, as the economy developed and urbanization accelerated5, farmland was rapidly lost and became fragmented. To tackle this issue, several policies were implemented to protect farmland, including the famous warning line of 120 million hectares (the minimum amount) of farmland raised in *the National General Land Use Planning* in 2007, and *the Basic Farmland Protection Regulation* released in 1998 (*Regulation* was strengthened in 2008). Furthermore, South Jiangsu Province changed the economic structure and the style of its economic expansion, so that further development of its economy would not be strongly reliant on the occupation of farmland. These policies decided the general trend of farmland morphological changes in this area.

### Suggestions for farmland protection

Avoiding fragmentation is a key aspect of farmland preservation^12^. China should reinforce the implementation of the “Agricultural land consolidation” program, including the merging of fragmented farmland patches. Additionally, a balance must be kept between farmland occupation and compensation, and the protection of basic farmland must also be implemented. From the perspective of landscape patterns, the protection of core farmland should be the first priority, alongside the reduction of boundary and branch farmland to keep the farmland shapes regular. Small islet farmland should be reduced or incorporated into the core farmland. Networks and the “corridors” of landscape are also essential for increasing the landscape’s connectivity^37^, implying that an increase in connector farmland could strengthen the *COHESION* of farmland landscape. With the current vigorous economic development, policy makers should not only protect high-quality and contiguous farmlands, but should also continue to promote the agricultural development, raise the level of agricultural modernization^34^, and adjust the relevant land use policies according to the local situation. Ideally, both the total amount and the morphological structure of farmland should remain balanced and stationary.

## Conclusions

This study analyzed farmland loss and farmland pattern changes using traditional landscape indices in South Jiangsu Province during 1985–2010. The increase in the *PD* and the decrease in the *NLSI*, and the *MESH* and *COHESION* indices demonstrated considerable farmland fragmentation. We also applied a new MSPA method that can visually present farmland patterns changes and guarantee the accuracy of farmland landscape analysis at the pixel level. Direct images showed a decrease in core farmland and an increase in other types of farmland, revealing that the most fragmented areas were in the northwest and the southeast of the study area. Overall, farmland in this area diminished and fragmented faster before 2008. By integrating a Markov chain model and the MSPA results, we could clearly observe the transition processes for the different farmland classes and how these transitions influenced (or aggravated) farmland fragmentation. We observed a relatively stable farmland configuration but obvious transitions to non-farmland. Results after 2000 indicated a faster and less deterministic progression towards a stationary farmland distribution. This situation had direct and significant relationships with socioeconomic, population, and policy factors, such as the regional *GDP* and the number of agricultural practitioners. Owing to the ecological and economic damage caused by fragmentation, high-quality and contiguous farmlands should be protected. From a landscape ecology perspective, scattered islet, boundary, and branch farmland should be reduced to protect the farmland shape and increase the connectivity of farmland in the study area.

Future studies should focus on farmland morphological changes after 2010, when South Jiangsu Province expedited economic restructuring that does not rely predominantly on industries that occupied large areas of farmland. Additionally, support vector machine (SVM) classification system could be employed in future studies to improve classification accuracy and reduce the amount of labor involved in classification.

## Methods

### Study area

**Figure 4:**
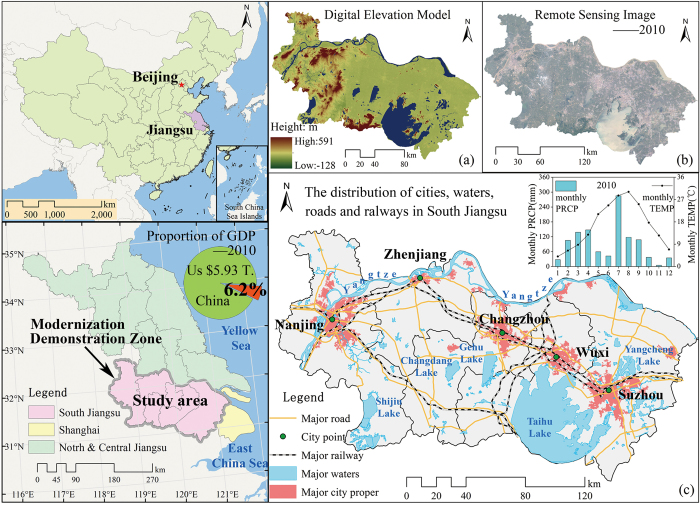
The study area (left), (**a**) an overall terrain view, (**b**) a remote sensing image (2010), and (**c**) a map showing major roads, railways, water bodies, and major cities in the study area. The map data was downloaded from http://www.geodata.cn and http://www.gscloud.cn/ (Remote Sensing Images, Administrative Boundaries, Digital Elevation Model and etc.). The figure was generated by Y.D. using ArcMap 10.0 (http://www.esrichina.com.cn/).

South Jiangsu Province (118.35°E–121.33°E, 30.76°N–32.61°N) is located in the southeast coastal region of China, spanning 5 cities (Nanjing, Zhenjiang, Changzhou, Wuxi, and Suzhou) and covering an area of 27,872 km^2^ (terrain: 50.45% plains, 28.4% hills, and 21.12% water). The region belongs to the East Asian monsoon climate zone, which is characterized by cold winters, hot summers, and a generally humid environment. The mean precipitation is 1000–1280 mm/yr, and in the rainy season (June and July), it is around 250 mm. The study area has rich water resources, including Lake Taihu (one of China’s five largest freshwater lakes), and the Yangtze River, which traverses the territory from east to west and is 432 km long (Fig. 4). The superior geographical location and climate of this region combine to provide an excellent environment for agricultural production.

The resident population of the region was 32.56 million in 2011, and the population density was 1166 people/km^2^, 8.3 times the national average. South Jiangsu has developed a unique regional economic development model: the South Jiangsu Model. This model is characterized by township and village enterprises, and is regarded as one of the most successful modes of rural territorial development in China. Therefore, the regional *GDP* reached 3.64 × 10^12^ Yuan, 6.4% of China’s total GDP. Per capita *GDP* reached 1.14 × 10^5^ Yuan in 2013, ranking 41^st^ in the world according to the International Monetary Fund (IMF). The region has seen some of the fastest growth in its economy and traffic network in China since the “Reform and Openness”, and its urbanization rate reached 70.8% in 2013. The South Jiangsu Modernization Demonstration Zone, the first (currently the only) regional zone taking modernization as its theme, was formally established in 2013.

South Jiangsu Province contained abundant farmland resources and the crop sowing area was 1.24 × 10^6^ ha in 2011. Although it is one of the main grain-producing areas in China, farmland area and food production (mainly rice with some wheat) have continued to decrease since the 1990s, resulting in a decrease in grain possession per capita of ca. 30%.

### Remote sensing data and data preprocessing

The remote sensing (RS) technique, as a mature Earth Observation (EO) technique, can provide rich ecological and geographical information and facilitate our understanding^38^,^39^,^40^. We obtained RS images for the years 1985, 1995, 2000, 2005, 2008, and 2010 from the US Geological Survey (http://earthexplorer.usgs.gov/). The image dataset was produced from the Landsat TM (Thematic Mapper) and ETM+ (Enhanced Thematic Mapper Plus) sensors, and the spatial resolution of all images was 0.09 ha/pixel (Supplementary Table S4). The starting point for this study was the initial period of the “Reform and Openness” program in the 1980s (1985, also the final year of the 6^th^ Five-Year Economic Plan) and the selected study years marked the ends of the 7–10^th^ Five-Year Economic Plan periods, allowing an assessment of the economic effects on farmland. The year 2008 was selected because (1) the economy developed faster during this year, which may have aggravated farmland morphological changes, and (2) the concept of permanent basic farmland was first introduced.

For each image, we performed image preprocessing using ENVI 4.7, which involves geometric correction, radiometric calibration, atmospheric correction, and image mosaicking and clipping. A few clouds were excluded by the Haze Tool (an IDL plug-in in ENVI). Then, we processed a maximum-likelihood supervised classification and included six main land use types: farmland, built-up land, forest, grassland, water bodies, and other land (Supplementary Figure S2). We assessed the classification accuracy using 300 random small ROIs (region of interest) selected from the land use data from *Data Sharing Infrastructure of Earth System Science* (http://www.geodata.cn/) and Google Maps. Our results from confusion matrices showed that the total accuracy of each classified image was around 87%, which was better than the recommended 85%^41^, and the kappa coefficient was around 0.7 (Table 4). After classification, we set farmland pixels as foreground and non-farmland (five other land-use types except farmland) pixels as background. Then we used the *Graphical User Interface for the Description of image Objects and their Shapes* (http://forest.jrc.ec.europa.eu/biodiversity/GUIDOS/) (GUIDOS) program to perform MSPA.

The main factors behind farmland changes may have been socioeconomic, cultural, and political trends^21^,^42^. We therefore obtained socioeconomic data from the Statistical Yearbook of each city (e.g. Nanjing Statistics Bureau website: http://www.njtj.gov.cn/) to study the driving forces behind farmland pattern changes during 1985–2010. The relevant factors chosen for this study were: (1) regional Gross Domestic Production (*GDP*), (2) regional Gross Agricultural Production (*GAP*), (3) proportion of *GAP* (*PGAP*), (4) total number of Practitioners (*P*), (5) Agricultural practitioners (*AP*), and (6) proportion of AP (*PAP*). A bivariate Pearson correlation analysis was used to investigate correlations between these factors, and landscape indices and morphological classes using SPSS 21.0.

### Landscape pattern indices

Landscape pattern indices can illustrate the relationship between landscape patterns and ecological processes at the landscape level^43^, and advance our understanding of the ecological functioning of landscapes^44^. Since the selection of specific indices for different landscapes appears idiosyncratic, and no individual index can capture the full complexity of spatial patterns, highly contingent and multiple sets of indices are required^45^. In this study, we selected the following four indices to reveal the morphological changes of farmland comprehensively: (1) the patch density (*PD*), (2) the normalized landscape shape index (*NLSI*), (3) the *COHESION* index (*COHESION*), and (4) the effective *MESH* size (*MESH*) These indices are calculated by FRAGSTATS^46^, and are defined and explained in Supplementary Table S5.

### Morphological spatial pattern analysis (MSPA)

MSPA is based on mathematical morphology and relies on two fundamental morphological operations: erosion and dilation^47^. MSPA can automatically segment preprocessed binary images at the pixel level into different types or classes representing different sizes, shapes, and connectivity^22^. This allows direct observation of the fragmentation process and the relationships between different classes, aiding the interpretation of results. MSPA uses five mutually exclusive types (or seven classes) for foreground pixels: core, islet, connector (including the bridge and loop classes), boundary (including the perforation and edge classes), and branch^22^. These types are defined in Table 2 and illustrated in Supplementary Figure S3.

MSPA is a highly scale-dependent approach, and so the relative abundance of MSPA classes is related to the density of the focal class (F), the level of contagion (H), the edge width of the morphological windows (*D*), and the connectivity rule (R)^48^. For each given map, F and H are set, so only changes in *D* and R will affect the MSPA results. R can be set to four neighbors (the pixels’ edges are directly connected to a central pixel) or eight neighbors (the pixels’ edges or corners are connected to a central pixel). The edge width (*D*), defined as 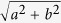 (*a* and *b* are the distance along the horizontal and vertical axes respectively), is used to determine the different classes in MSPA. R and *D* determine the quantity of each class, but only R decides the general trends of the relative proportions and shapes of different classes^49^. A smaller value of *D* can provide more differentiation among structural classes and more information on landscape patterns^50^.

During 1985–2010, the proportions of farmland area (F) in South Jiangsu ranged from 49% to 65%, and the contagion index (H) was around 47–50. According to an experimental analysis of random maps with contagion and of real maps^48^, we chose a connectivity rule (R) set to 8 neighbors, and set *D* = 4 (i.e., edge width = 120 m) for this study. According to the characteristics of different classes, a decrease in the amount of core and connector farmland, and an increase of other types can reveal the fragmentation directly.

### Markov chain models

Markov chain models can analyze temporal landscape changes^51^, and have been used previously in landscape ecology studies to identify the direction and rate of the transition between patches, corridors, and matrices^52^,^53^. In this study, we used discrete-time, finite-state, homogeneous (stationary) Markov chain models to analyze the succession of MSPA classes in 1985–2000 and 2000–2010. Our model viewed the landscape as a large set of pixels, and the state of each pixel was identified by the occupying land use type (Supplementary Panel S1). After obtaining the transition probability matrix **P**, we used two variables^52^ to analyze the matrices: the convergence rate *ρ* and the entropy H(**P**). A smaller *ρ* indicates a slower convergence rate to a stationary distribution. If H(**P**) is closer to 0, this indicates a more deterministic Markov transition, and values closer to 1 indicate a more random Markov transition.

## Additional Information

**How to cite this article**: Cheng, L. *et al.* Analysis of farmland fragmentation in China Modernization Demonstration Zone since "Reform and Openness": a case study of South Jiangsu Province. *Sci. Rep.* **5**, 11797; doi: 10.1038/srep11797 (2015).

## Supplementary Material

Supplementary Information

## Figures and Tables

**Table 1:** Changes in farmland area and relative proportion in South Jiangsu Province from 1985 to 2010.

**Years**	**Total area(km**2)	**Farmland area(km**2)	**Proportion of farmland(F)**	**Average annual decrement (km**2**/yr)**	**Average annual decrement rate(%/yr)**
1985	27749.26	17915.52	64.56%	N/A	N/A
1995	27749.26	16959.94	61.12%	−95.56	−0.53%
2000	27749.78	16397.31	59.09%	−112.53	−0.66%
2005	27749.77	15194.71	54.76%	−240.52	−1.47%
2008	27778.31	14024.83	50.49%	−389.96	−2.57%
2010	27778.27	13638.64	49.10%	−193.09	−1.38%

**Table 2:** Explanations of the five types and definitions for the seven classes used in the MSPA for this study.

**Type**	**Explanation**	**Class**	**Definition**
Core	“**Matrix**” (main, stable and contiguous) farmland	Core	Foreground pixels whose distance to the background is greater than the given edge width *D*
Islet	“**Patch**” (interspersed) farmlands unsuitable for mass production	Islet	Foreground connected components that do not contain any core pixels
Connector	“**Corridor**” farmland that connect different or same core farmland	Bridge	Pixels that emanate from two or more core connected components
		Loop	Pixels that emanate from the same core connected component
Boundary	“**Ambient**” farmland around the core farmland that separates core farmland area from non-farmlands area	Perforation	Pixels within a distance *D* to a hole (foreground pixels that separate foreground from background which is inside the foreground pixels) → inner boundaries
		Edge	Pixels whose distance to a core pixel is lower than or equal to *D* and that face the background without holes → outer boundaries
Branch	“**Discrete**” farmland randomly attached to the boundary farmland	Branch	Pixels that do not belong to any of the previously defined classes

**Table 3:** The Pearson correlation between population and economic factors, and changes in landscape indices and morphological classes during 1985–2010.

**PearsonCorrelation**	**Morphological classes**							**Landscape indices**			
	**C**	**I**	**BE**	**L**	**P**	**E**	**BH**	***PD***	***CO***	***MESH***	***NLSI***
*GDP*	−0.918^**^				−0.972^**^	0.947^**^	0.965^**^		−0.993^**^	−0.978^**^	−0.977^**^
*GAP*	−0.950^**^				−0.941^**^	0.959^**^	0.950^**^		−0.944^**^	−0.982^**^	−0.961^**^
*PGAP*	0.961^**^		−0.944^**^			−0.940^**^					
*TP*	−0.828^*^								−0.970^**^	−0.927^**^	−0.970^**^
*AP*	0.968^**^		−0.953^**^			−0.957^**^	−0.931^**^			0.951^**^	
*PAP*	0.961^**^		−0.944^**^		0.933^**^	−0.960^**^	−0.948^**^			0.970^**^	0.954^**^
N	6		6		6	6	6		6	6	6

Abbreviations: Core (C), Islet (I), Bridge (BE), Loop (L), Perforation (P), Edge (E), Branch (BH).^**^Correlation is significant at the 0.01 level (2-tailed). Blank entries denote no significant relationships.

**Table 4:** Total classification accuracy (TCA), kappa coefficient, contagion index (H), and proportion of farmland (F) for interpretation of the 1985–2010 data.

**Year**	**TCA(farmland)**	**Kappa Coefficient**	**Contagion Index(0** **≤** **H** **≤** **100)**	**Proportion of Farmland (F)**
1985	0.85	0.70	49.579	64.56%
1995	0.87	0.73	48.702	61.12%
2000	0.86	0.71	47.088	59.09%
2005	0.87	0.74	47.246	54.76%
2008	0.88	0.75	47.547	50.49%
2010	0.87	0.73	47.463	49.10%
