# Supplementary material for: Analysis of farmland fragmentation in China Modernization Demonstration Zone since “Reform and Openness”: a case study of South Jiangsu Province
Source: Sci Rep. 2015 Jul 2;5:11797. doi: 10.1038/srep11797 (PMC4488744; doi:10.1038/srep11797)
Supplement: Supplementary Information [file srep11797-s1.docx]

**Supplementary Information**

**Analysis of farmland fragmentation in China Modernization Demonstration Zone since “Reform and Openness”: a case study of South Jiangsu Province**

*Authors:* Liang Cheng^1, 2, 3, 4^, Nan Xia^1, 3^, Penghui Jiang^1, 3^*, Lishan Zhong^1, 3^, Yuzhe Pian^1, 3^, Yuewei Duan^1, 3^, Qiuhao Huang^1, 2, 3, 4^, Manchun Li^1, 2, 3, 4^**

^1^ Jiangsu Provincial Key Laboratory of Geographic Information Science and Technology, Nanjing University, Nanjing, 210093, China

^2^ Collaborative Innovation Center for the South Sea Studies, Nanjing University, Nanjing 210093, China

^3^ Department of Geographic Information Science, Nanjing University, Nanjing 210093, China

^4^ Collaborative Innovation Center of Novel Software Technology and Industrialization, Nanjing University, Nanjing, China

**
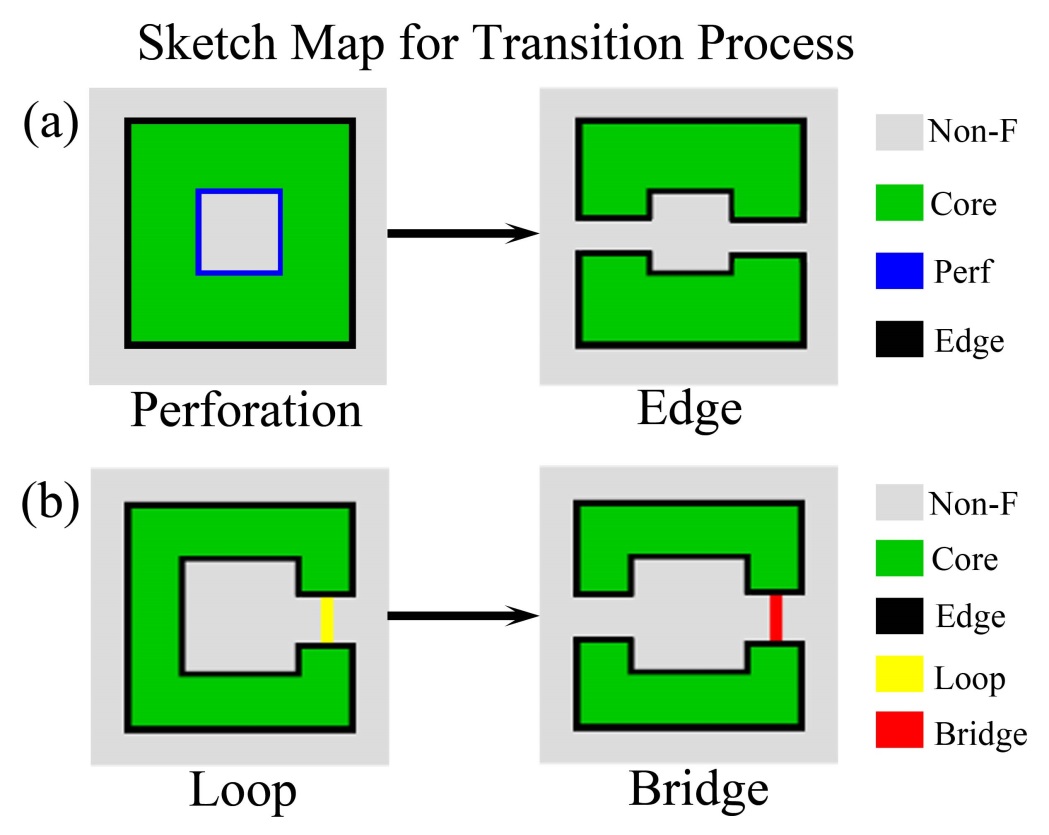
**

**Supplementary Figure S1.** Sketch map for transition process from the perforation to edge farmlands (a), and from the loop to bridge farmlands (b). (Abbreviation: Non-F, Non-farmland; Perf, Perforaion). These two kinds of transition obviously show that farmlands become more fragmented: (a) The farmland becomes two patches when inner boundary (perforation) becomes outer boundary (edge); (b) The connector farmland which links the same core (loop) becomes connector farmland that links different two patches of core farmland (bridge) when farmland becomes fragmented.

The figure was generated by L.C. and N.X using ArcMap 10.0 (<http://www.esrichina.com.cn/> ).

**
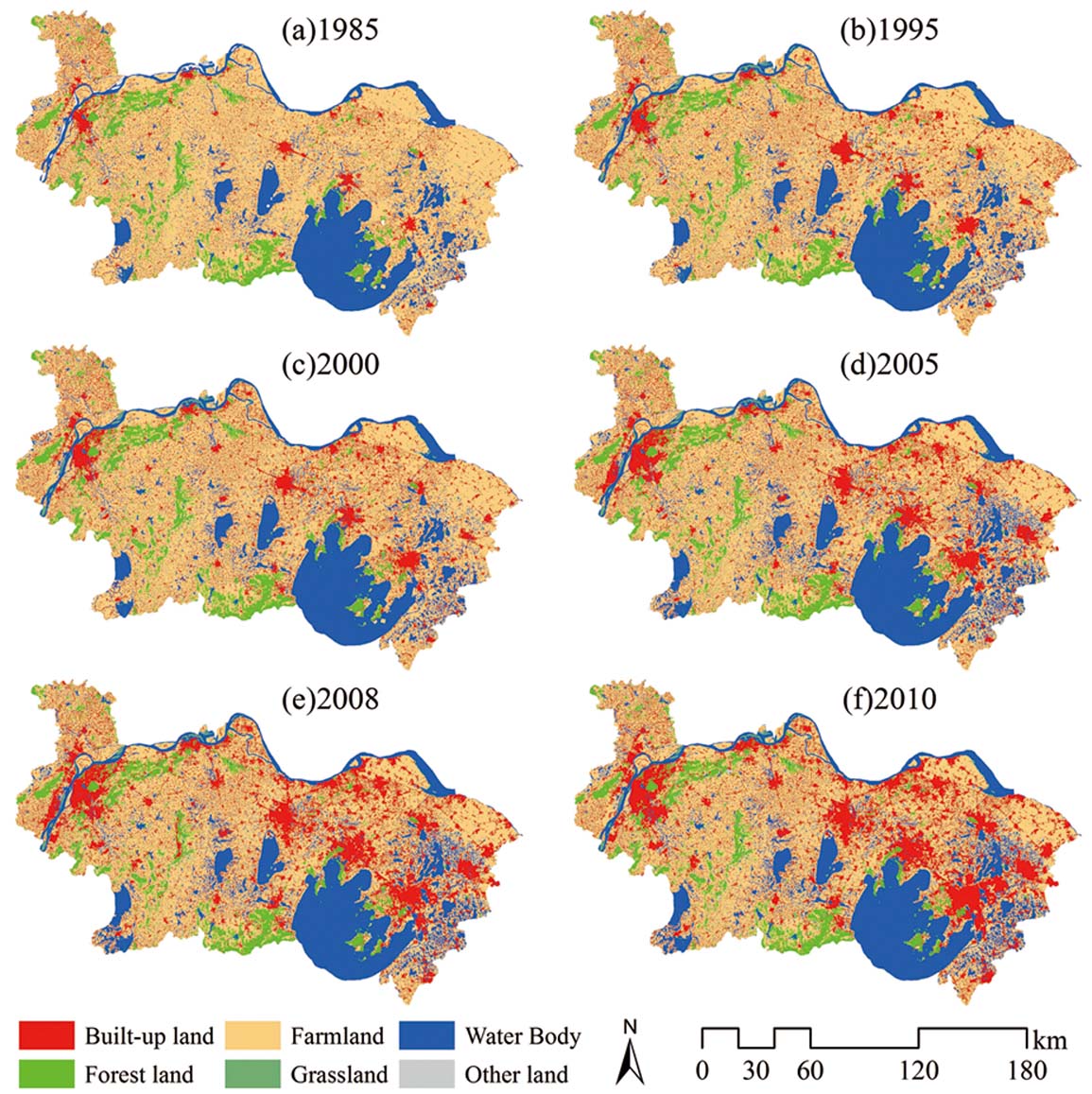
**

**Supplementary Figure S2.** Maximum-likelihood supervised classification maps showing land use change in South Jiangsu Province, China for (a) 1985, (b) 1995, (c) 2000, (d) 2005, (e) 2008, and (f) 2010.

The figure was generated by L.C. and N.X using ArcMap 10.0 (<http://www.esrichina.com.cn/> ).

**
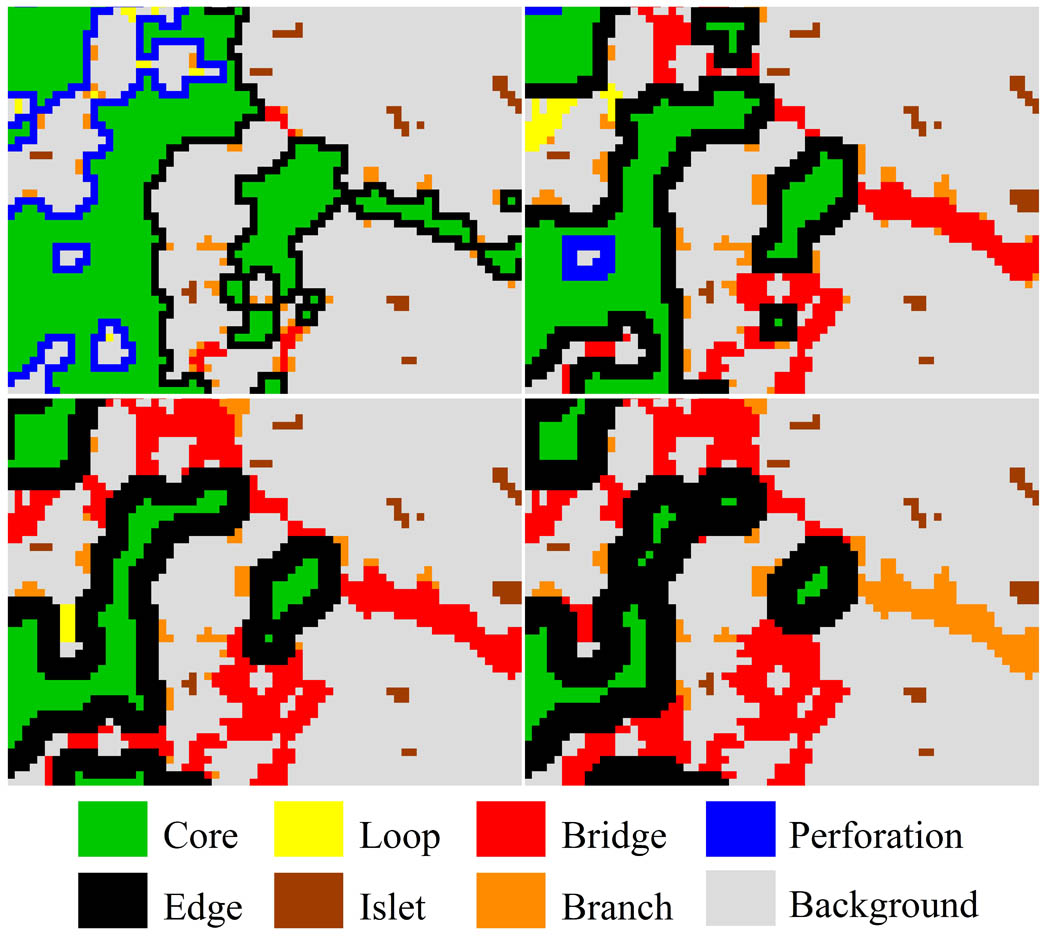
**

**Supplementary Figure S3.** Illustrations of seven MSPA classes for edge width = 30 m (top left), 60 m (top right), 90 m (bottom left), and 120 m (bottom right). Different edge width will greatly influence the morphology of different classes.

The figure was generated by L.C. and N.X using ArcMap 10.0 (<http://www.esrichina.com.cn/>).

**Supplementary Table S1.** The transition matrix for the Markov chain model showing the probability of a pixel transition from a 1985 MSPA class or non-farmland (columns) to a 2000 MSPA class or non-farmland (rows).

| 2000 Mar-  -kov state | 1985 Markov State | | | | | | | |
| --- | --- | --- | --- | --- | --- | --- | --- | --- |
|  | C | I | P | E | L | BE | BH | NF |
| C | **0.8186** | 0.0003 | 0.0236 | 0.0203 | 0.0128 | 0.0121 | 0.0003 | 0.0001 |
| I | 0.0005 | **0.4353** | 0.0005 | 0.0031 | 0.0009 | 0.0028 | 0.0094 | 0.0001 |
| P | 0.0421 | 0.0000 | **0.5737** | 0.0074 | 0.0443 | 0.0083 | 0.0089 | 0.0028 |
| E | 0.0418 | 0.0100 | **0.1816** | **0.7366** | 0.0442 | 0.0476 | 0.0343 | 0.0039 |
| L | 0.0114 | 0.0057 | 0.0426 | 0.0133 | **0.5408** | 0.0400 | 0.0058 | 0.0009 |
| BE | 0.0161 | 0.0000 | 0.0353 | 0.0533 | **0.1861** | **0.7305** | 0.0158 | 0.0015 |
| BH | 0.0057 | 0.0478 | 0.0174 | 0.0276 | 0.0284 | 0.0318 | **0.6139** | 0.0030 |
| NF | **0.0639** | **0.5009** | **0.1253** | **0.1384** | **0.1424** | **0.1270** | **0.3114** | **0.9877** |
| SUM | 1.0000 | 1.0000 | 1.0000 | 1.0000 | 1.0000 | 1.0000 | 1.0000 | 1.0000 |
| C–Core; I–Islet; P–Perforation; E–edge; L–Loop; BE–Bridge; BH–Branch; NF–Non-farmland  Bold values indicate that values are larger than 0.05 | | | | | | | | |
| Convergence rate: *ρ* **=** 1.1647 Normalized entropy: **H(P) =** 0.4093 | | | | | | | | |

**Supplementary Table S2.** The transition matrix for the Markov chain analysis showing the probability of a pixel transition from a 2000 MSPA class (columns) or non-farmland to a 2010 MSPA class or non-farmland (rows).

| 2010 Mar-  -kov state | 2000 Markov State | | | | | | | |
| --- | --- | --- | --- | --- | --- | --- | --- | --- |
|  | C | I | P | E | L | BE | BH | NF |
| C | **0.8146** | 0.0013 | 0.0236 | 0.0201 | 0.0141 | 0.0134 | 0.0129 | 0.0069 |
| I | 0.0003 | **0.5148** | 0.0006 | 0.0020 | 0.0013 | 0.0014 | 0.0133 | 0.0001 |
| P | 0.0095 | 0.0000 | **0.5080** | 0.0071 | 0.0113 | 0.0029 | 0.0016 | 0.0005 |
| E | 0.0374 | 0.0071 | **0.2690** | **0.6657** | 0.0304 | 0.0401 | 0.0168 | 0.0039 |
| L | 0.0029 | 0.0000 | 0.0210 | 0.0036 | **0.5884** | 0.0190 | 0.0020 | 0.0002 |
| BE | 0.0104 | 0.0027 | 0.0301 | 0.0366 | **0.1791** | **0.6759** | 0.0075 | 0.0009 |
| BH | 0.0063 | 0.0070 | 0.0177 | 0.0273 | 0.0259 | 0.0357 | **0.6782** | 0.0010 |
| NF | **0.1186** | **0.4670** | **0.1301** | **0.2376** | **0.1494** | **0.2117** | **0.2677** | **0.9864** |
| SUM | 1.0000 | 1.0000 | 1.0000 | 1.0000 | 1.0000 | 1.0000 | 1.0000 | 1.0000 |
| C–Core; I–Islet; P–Perforation; E–edge; L–Loop; BE–Bridge; BH–Branch; NF–Non-farmland  Bold values indicate that values are larger than 0.05 | | | | | | | | |
| Convergence rate: *ρ* **=** 1.2256 Normalized entropy: **H(P) =** 0.3848 | | | | | | | | |

**Supplementary Table S3.** The regional *GDP* (Gross Domestic Production), regional *GAP* (Gross Agricultural Production), proportion of *GAP*, practitioners, agricultural practitioners (AP), and proportion of AP during 1985-2010.

| Years | Regional *GDP*  (10^8^*yuan*) | Regional *GAP*  (10^8^*yuan*) | Proportion of *GAP* | Practitioners  (10^4^) | Agricultural  Practitioners  (10^4^) | Proportion  of *AP* |
| --- | --- | --- | --- | --- | --- | --- |
| 1985 | 432.68 | 62.43 | 14.43% | 1156.31 | 402.29 | 34.79% |
| 1995 | 2894.77 | 241.34 | 8.34% | 1212.91 | 287.46 | 23.70% |
| 2000 | 4814.67 | 273.35 | 5.68% | 1134.16 | 284.77 | 25.11% |
| 2005 | 11589.06 | 344.14 | 2.97% | 1389.53 | 188.25 | 13.55% |
| 2008 | 19108.48 | 494.6 | 2.59% | 1676.31 | 165.22 | 9.86% |
| 2010 | 25067.38 | 584.32 | 2.33% | 1899.79 | 164.11 | 8.64% |

**Supplementary Table S4.** Data sources of farmland landscape information for the study area

| Year | Data source & Remote sensing image | | | |
| --- | --- | --- | --- | --- |
|  | 119/038 | 119/039 | 120/037 | 120/038 |
| 1985 | 1984.08.04, TM | 1984.08.04, TM | 1985.05.07, TM | 1985.05.07, TM |
| 1995 | 1995.12.09, TM | 1995.12.09, TM | 1994.07.22, TM | 1994.07.22, TM |
| 2000 | 2000.12.06, TM | 2000.11.04, TM | 2000.04.17, TM | 2000.04.17, TM |
| 2005 | 2005.10.17, TM | 2005.10.17, TM | 2005.10.24, TM | 2005.10.24, TM |
| 2008 | 2008.04.22, ETM+ | 2008.04.22, ETM+ | 2009.10.03, TM | 2008.04.22, ETM+ |
| 2010 | 2010.05.24, TM | 2010.05.24, TM | 2010.08.19, TM | 2010.08.19, TM |

**Supplementary Table S5.** Landscape indices and explanations

| Indices | Definitions and Explanations | | | |
| --- | --- | --- | --- | --- |
| *PD* |  | *n_i_* =number of patches in the landscape of patch type i  *A* = total landscape area (m^2^) | | |
|  | *PD* is multiplied by 10,000 and 100 to convert to 1 km^2^ (100 hectares). *PD* equals the number of patches in a unit landscape area, and is a straightforward measure of fragmentation. Larger *PD* may indicate that the landscape was more fragmented. | | | |
| *NLSI* | $\frac{e_{i} -\min e_{i} }{\max e_{i} -\min e_{i} }$ | e_i_ = total length of edge (or perimeter) of class i in terms of number of cell surfaces  max e_i_ = maximum total length of class i  min e_i_ = minimum total length of class i | | |
|  | *NLSI* is the normalized version of the landscape shape index (range = 0–1), and indicates whether the patch type is relatively rare (*NLSI* < 0.1) or relatively dominant (*NLSI* > 0.5). | | | |
| *COHESION* |  | | | pij* = perimeter of patch ij in terms of number of cells  aij* = area of patch ij in terms of number of cells  *Z* = total number of cells in the landscape. |
|  | *COHESION* is sensitive to the aggregation of the focal class, and increases as the patches become more clumped or aggregated in distribution. That means a smaller *COHESION* may indicate a more fragmented landscape. | | | |
| *MESH* |  | | a_ij_ = area (m^2^) of patch ij  *A* = total landscape area (m^2^) | |
|  | MESH is divided by 10,000 to convert to hectares, and has proven to monotonically decrease with increasing fragmentation, and is consistent throughout the fragmentation process^1^. | | | |
| Further details and the mathematical foundation of these indices can be found in FRAGSTATS help^2^ | | | | |

1. Jaeger, J. A. G. Landscape division, splitting index, and effective MESH size: new measures of landscape fragmentation. *Landsc. Ecol.* **15**:115-130 (2000).

2. McGarigal, K., *FRAGSTATS HELP*. (University of Massachusetts, 2002) Available at: <http://www.umass.edu/landeco/research/fragstats/documents/fragstats.help.4.2.pdf>. (Accessed: 14th March 2015).

**Supplementary Panel S1.** The principle of Markov chain model for MSPA classes in this study

The model for this study included two main possible pixel states: farmland and non-farmland. The possible state of each farmland pixel was identified by its unique MSPA class attribute. There are 3 possible pixel transitions: (1) transitions between two different MSPA classes; (2) MSPA classes to non-farmland; (3) non-farmland to MSPA classes.

**1.** Transition matrix **P**

Suppose that there are *n* possible states. Let **X(t)** ∈ {1, 2,…, *n*} denote the state at time t and **X(t + 1)** ∈ {1, 2,…, *n*} at time t + 1. Let **P** be an *n* × *n* matrix of transition probabilities, whose elements, P_ij_, are the conditional probabilities:

P_ij_ = **P**[**X(t + 1)** ∈ *i* | **X(t)** ∈ *j*], with *i*, *j* = 1,2,…,*n*. (1)

The i^th^ column of **P** indicates the Markov state at time t, and the j^th^ row indicates the Markov state at time t + 1. If **X(t)** is the 1 × *n* vector of probabilities that a pixel is in state i, then

**X(t+1)** = **P****X(t)** (2)

The diagonal elements in the transition matrix **P** represent the probabilities of a certain pixel state (MSPA class or non-farmland) persisting, while the off-diagonal elements represent the probabilities of changes between different pixel states.

**2.** Convergence rate *ρ*

The dominant eigenvector of **P** can estimate the rate of convergence from the initial state, **X(t)**, to the asymptotic and stationary (equilibrium) distribution, **X(e)**. Convergence rate can be measured by the damping ratio (*ρ*):

*ρ* = ω_1_/|ω_2_| (3)

where ω_1_ and ω_2_ are the first and second eigenvalues (found using MATLAB) of **P**, normalized to sum to 1. Smaller values of *ρ* indicate a slower convergence rate to **X(e)**.

**3.** The entropy of **P**

The entropy of the each column i of **P**, as values’ relative magnitudes, should be taken into account. The entropy is an inverse measure of the predictability of successional changes, and each morphological class in column i (**H_i_**) can be denoted as

**H_i_** = –Σ_j_ (P_ij_ × log(P_ij_)) (4)

and the normalized [0, 1] entropy for all *n* states of the Markov chain was

H(**P**) = Σ_i_ (ω_i_ × **H_i_**) / log(*n*) (5)

where *n* is the total number of all morphological classes (i.e., the number of columns in **P**). Values of H(**P**) closer to 0 indicated a more deterministic Markov transition, and values closer to 1 indicated a more random Markov transition.
